# Exercise and cancer: a position statement from the Spanish Society of Medical Oncology

**DOI:** 10.1007/s12094-020-02312-y

**Published:** 2020-02-13

**Authors:** M. Pollán, S. Casla-Barrio, J. Alfaro, C. Esteban, M. A. Segui-Palmer, A. Lucia, M. Martín

**Affiliations:** 1 Cancer and Environmental Epidemiology, Carlos III Institute of Health, Madrid, Spain; 2 Consortium for Biomedical Research in Epidemiology and Public Health (CIBERESP), Madrid, Spain; 3 Exercise-Oncology Unit, Spanish Cancer Association, Madrid, Spain; 4 GEICAM (Spanish Breast Cancer Group), Madrid, Spain; 5 Medical Oncology, Hospital de Terrassa, Barcelona, Spain; 6 Medical Oncology, Hospital Virgen de la Salud, Toledo, Spain; 7 Medical Oncology, Parc Taulí Hospital Universitari, Sabadell, Spain; 8 Faculty of Sport Sciences, Universidad Europea de Madrid, Calle Tajo, s/n, 28670 Villaviciosa de Odón, Madrid, Spain; 9 Instituto de Investigación Hospital 12 de Octubre and CIBER de Envejecimiento Saludable y Fragilidad (CIBERFES), Madrid, Spain; 10 Instituto de Investigacion Sanitaria Gregorio Marañón, Universidad Complutense, Madrid, Spain; 11 Centro de Investigación Biomédica en Red de Cáncer (CIBERONC), Madrid, Spain; 12 Medical Oncology Service, Hospital General Universitario Gregorio Marañón, Calle Dr. Esquerdo 46, 28007 Madrid, Spain

**Keywords:** Oncology, Cancer, Exercise, Physical activity, Exercise-oncology

## Abstract

Due to improvements in the number of cancer survivors and survival time, there is a growing interest in healthy behaviors, such as physical activity (PA), and their potential impact on cancer- and non-cancer-related morbidity in individuals with cancer. Commissioned by the Spanish Society of Medical Oncology (SEOM), in this review, we sought to distill the most recent evidence on this topic, focusing on the mechanisms that underpin the effects of PA on cancer, the role of PA in cancer prevention and in the prognosis of cancer and practical recommendations for clinicians regarding PA counseling. Despite the available information, the introduction of exercise programs into the global management of cancer patients remains a challenge with several areas of uncertainty. Among others, the most effective behavioral interventions to achieve long-term changes in a patient’s lifestyle and the optimal intensity and duration of PA should be defined with more precision in future studies.

## Introduction

Regular and adequate physical activity (PA) is associated with key benefits to human health, such as improvements in weight control, muscular and cardiorespiratory fitness, bone and functional health and a reduced risk of falls and several noncommunicable diseases, including diabetes, cardiovascular disease, depression and some cancers [1].

Due to improvements in the management of cancer, the number of cancer survivors and survival time are increasing. Consequently, interest in healthy behaviors, such as PA, and their potential impact on cancer- and non-cancer-related morbidity in these individuals has rapidly increased [2].

Commissioned by the Spanish Society of Medical Oncology (SEOM), in this review, we sought to distill the most recent evidence on this topic, focusing on the mechanisms that underpin the effects of PA on cancer, describing the role of PA in cancer prevention and prognosis, and providing practical recommendations to clinicians on managing PA counseling.

## Biological mechanisms underpinning the potential anticancer effects of exercise

Of note, PA is any bodily movement produced by skeletal muscles that requires energy expenditure, whereas exercise is a subset of PA that is planned, structured and repetitive and that has a final or an intermediate objective of improving or maintaining physical fitness. The epidemiological evidence regarding the risk of some cancers mainly refers to regular PA (usually self-reported, i.e., through questionnaires). Thus, regular exercise or “exercise training” is a proxy, but not a perfect surrogate, for PA and is thought to induce more profound molecular adaptations than PA.

There is growing evidence from preclinical research that regular exercise can influence cancer development or the rate of tumor growth once malignancy has initiated. For instance, a recent meta-analysis of 28 preclinical studies in breast tumors (*n* = 2085 animals) found large favorable effects for exercise training on proliferation and apoptosis [3]. Exercise is also emerging as a potential coadjuvant treatment; when combined with cyclophosphamide, exercise delays murine breast tumor growth versus chemotherapy alone [4], and similar findings have been reported for exercise combined with anthracyclines [5–7]. There is, however, heterogeneity among studies in the tumor models used, ranging from tumor transplant (where there is also substantial variability, e.g., syngeneic versus xenograft models) to carcinogen-induced or genetically engineered mouse models [8]. The type of exercise training to which mice are typically subjected to before/upon tumor inoculation also varies between studies (forced treadmill, forced swimming, voluntary wheel running). The duration of exercise in the trials typically consists of several weeks (~ 4 to 10 weeks), which can be translated to human “years.”

Exercise can have an impact on tumor development, growth or dissemination through several mechanisms. First, exercise might help to prevent cancer by reducing the circulating levels of several mediators, such as insulin growth factor-1 (IGF-1) [9–14], a mitogen that triggers cell proliferation [15]. Exercise can also reduce the levels of hyperphosphorylated retinoblastoma protein (Rb) in a chemically induced rat model of mammary carcinogenesis [16, 17], increase ß-catenin phosphorylation in colon polyps [18, 19], and reduce the levels of micro-RNA 21 [20].

Exercise can upregulate tumor suppressors, such as the tumor suppressor programmed cell death protein 4 in a murine model of estrogen receptor-positive breast cancer (BC) [21]. In addition, exercise-induced catecholamines might reduce BC development through activation of the Hippo tumor suppressor pathway [22] and exercise-induced increased p53 activation, leading to tumor prevention, as shown in mouse models of skin [13, 23] and lung [24] cancer.

Exercise training can stimulate apoptosis, as shown in xenograft models of lung adenocarcinoma [24] and human pancreatic and prostate cancers [25, 26] and in murine models of skin tumorigenesis [12] and mammary carcinoma [4, 16, 27]. Exercise also exerts proapoptotic effects on cultured prostate cancer cells [28], estrogen receptor-positive BC cells [29, 30] and lymph node metastases of prostate tumor cells [25]. Exercise additionally reduces the levels of the antiapoptotic protein B cell lymphoma 2 [16, 31] and stimulates the proapoptotic proteins Bax and Bak [4, 16, 24] and the protein kinase AMPK [32, 33].

Hypoxia and poor blood supply promote an aggressive cancer phenotype and contribute to ineffective systemic therapy [34]. In this respect, exercise may promote a shift toward a more “normalized” tumor microenvironment by improving intratumoral perfusion/vascularization, at least in orthotopic murine models of human BC [4, 35] and prostate cancer [36–38] and in xenografts of different tumors (melanoma, pancreas) [39].

Exercise might also attenuate the development of metastases. Mouse exercise training can decrease catenin while increasing E-cadherin inside tumors [19, 40]. Importantly, cadherins act as glue between epithelial cells, and their loss can favor malignancy by allowing the disaggregation of cells, which can then invade locally or metastasize [40]. Moderate-intensity mouse swimming can suppress liver cancer metastases via boosting the activity of dopamine receptor 2 [40]. Exercise may also modulate blood–brain barrier integrity by maintaining the expression levels of occludin or claudin-5 proteins [41], thereby preventing metastatic progression to the brain [42]. On the other hand, inflammatory cells within the tumor microenvironment supply bioactive molecules that sustain cancer hallmarks [43–45]. In this context, mouse exercise training decreases macrophage infiltration in allogeneic lymphoid tumors [46], Ehrlich tumor cells [47] and colon polyps [18].

One major potential “anticancer” effect of exercise lies in an enhancement of immune function [43, 48]. At moderate intensities, exercise can stimulate the innate immune system, especially natural killer (NK) cells [49, 50]. A 6-week mouse wheel running program had preventive effects against the development of several types of tumors (melanoma, liver and lung cancers), and the effect was mediated by improved NK cell infiltration into the tumors, which in turn was mediated by the enhanced tumor expression of ligands for several NK cell-activating receptors [51]. A previous study showed that exercise training increased the cytolytic capacity of resident peritoneal macrophages against mastocytoma cells [52]. Mouse exercise training could also polarize the immunological response toward an efficient “antitumor” macrophage profile 1, which is linked to the production of T-helper 1 cytokines [52–56]. Short-term (6-day) moderate exercise before the injection of melanoma cells into mice decreased their metastatic spread, which was partially mediated by increased antitumor macrophage cytotoxicity [57]. Preliminary data from mice [53, 58–60] and cancer patients suggest that exercise training may help to reduce the immunosuppressive effects of T regulatory lymphocytes [61]. Finally, regular exercise can increase alveolar macrophage antitumor cytotoxicity in vitro, which would mediate a protective effect against mouse lung metastases [62, 63].

Importantly, skeletal muscle, especially during contractions, releases molecules collectively known as “myokines” into the bloodstream, which act systemically to induce a myriad of health-promoting effects, such as decreased inflammation and reduced insulin resistance [64]. Some myokines might also induce direct anticancer effects (via the stimulation of apoptosis in tumor cells), such as oncostatin M in hormone-sensitive BC [30] or secreted protein acidic and rich in cysteine (SPARC, also known as osteonectin) in colon cancer [65]. The aforementioned exercise-induced infiltration of NK cells into tumors seems to be mediated by the release of interleukin 6 by muscle into the bloodstream [49, 51, 66].

## Physical activity and cancer prevention

According to the World Health Organization (WHO), up to 31% of the adult population worldwide and 35% in Europe are physically inactive [67]. PA is difficult to measure for the following reasons: (1) there are at least four domains: occupational, household, transportation and leisure time; (2) PA questionnaires on past and current activity are subject to recall bias; and (3) objective methods (pedometers or accelerometers) can only be used in prospective studies for short time periods, and they may not always represent overall PA. Fortunately, smartphones and other devices now allow PA monitoring and will hopefully provide more accurate measures in the future.

Body mass is related to PA and cancer risk, acting as a confounder. However, the prevention of adiposity may mediate the relationship between PA and cancer, and controlling for adiposity could lead to underestimating the real effect of PA [68].

Of note, 1 metabolic equivalent (MET) is the rate of energy expenditure while resting or 3.5 ml O_2_/kg body weight/min on average. Moderate PA (e.g., brisk walking) usually requires an energy expenditure of 3–6 MET, whereas vigorous PA (e.g., jogging) requires an energy expenditure above 6 MET. The WHO recommends that adults engage in ≥ 150 min/week of moderate PA or ≥ 75 min/week of vigorous PA or a combination thereof. If a person does an ~ 3 MET activity (e.g., brisk walking on a level surface) for 1 h, he or she has done 3 MET-hours of PA. If this person does this same PA on every day of the week, he/she has done 21 (= 3 × 7) MET-hours/week. If a person does an ~ 8 MET activity (e.g., jogging) for 1 h on each day of the week, he/she has done 56 (= 8 × 7) MET-hours/week.

The World Cancer Research Fund and the American Institute for Cancer Research periodically publish the conclusions of a panel reviewing evidence linking food, nutrition and PA with cancer risk [69]. The evidence is classified as follows: (1) convincing: available results support a causal relationship; (2) probable: evidence supports a probable causal relationship; and (3) limited: results are not considered sufficient to rate the relationship as convincing or probable. In the last category, a distinction is made between limited-suggestive evidence when an effect is reported but there were methodological problems and limited-not conclusive evidence when there were insufficient data and/or the results were too heterogeneous. The panel concluded that regular, sustained PA protects against several types of cancer independent of body fat [69]. This evidence comes from high-income countries and is mainly based on leisure-time PA. The three tumors with the most solid results are colon, postmenopausal BC and endometrial.

### Colorectal cancer

The evidence for colon cancer is judged as “convincing,” with an overall risk reduction of approximately 20% in the most physically active group compared with the less active group [70]. The effect is weaker or absent for rectal cancer. However, a pooled analysis of 12 prospective cohort studies with information on leisure-time PA at baseline compared the group at the 90th percentile of PA with the group under the 10th percentile and showed a reduced incidence of both colon (13% reduction) and rectal cancer (12%) after controlling for body mass index (BMI) [71]. Regarding the amount of PA required to obtain maximum benefit, a cohort of more than 40,000 men in the USA (The Health Professionals Follow-up Study) showed that aerobic PA seems to be more beneficial and that overall PA is more relevant than the intensity of PA [72]. Finally, while a benefit was observed in men meeting current guidelines (17% risk reduction), the maximum benefit (32% risk reduction) was observed for PA ≥ 30 metabolic equivalents (MET)-hours/week, which is equivalent to 10 h or more of walking/week [72]. A meta-analysis evaluating the dose–response shape of PA for different endpoints, including colon cancer, showed that major gains occurred at lower levels of activity (up to 50 MET-hours/week), while a decrease in risk was minimal at levels higher than 50–65 MET-hours/week [73].

### Breast cancer

The evidence for postmenopausal BC is judged as “probable” [74]. Most studies show a protective effect with a 13% decreased risk in high versus low PA groups [74]. For recreational PA, a nonlinear dose–response was observed with a greater decrease in the risk for PA activity at > 20 MET-hours/week [74]. The pooling analysis with information on baseline leisure PA showed a reduction of 7% in the incidence of BC between the 90th and the 10th percentiles of PA [71]. Evidence for PA in premenopausal BC was rated as “limited-suggestive” for total PA and as “probable” for vigorous-intensity PA [74]. In Canada, a cohort study with 39,000 women reported a clear downward trend of BC incidence based on the number of MET-hours/week, which was mainly due to the risk reduction observed for premenopausal tumors [75]. Finally, a case–control study in Spain showed a reduced risk of 5% per 6 MET-hours/week [76]. The protection was particularly important for nulliparous women (12% risk reduction per 6 MET-hours/week) [76].

### Endometrial cancer

The evidence for endometrial cancer was rated as “probable,” and the results showed a lower risk of endometrial cancer with higher levels of PA [77]. A meta-analysis reported a 20% risk reduction in high versus low PA groups [78]. This inverse association was only observed in overweight/obese women [78]. The pooled analysis of PA at baseline in 12 cohorts showed a risk reduction of 21% between the two extreme deciles of PA before taking BMI into account, while adjusting for BMI reduced the benefit to a nonsignificant risk reduction of 2% [71]. In the stratified analyses, PA was only associated with endometrial cancer in women with a BMI equal to or greater than 25 [71].

### Lung cancer

A recent report classifies the evidence for lung cancer as “limited-suggestive” [69]. Leisure-time PA was considered in a systematic review, showing a clear inverse association with all histological lung cancer subtypes but only among former or current smokers [79]. The pooled analysis of cohort studies on leisure-time PA reported a 27% reduction in lung cancer incidence in the highest decile of PA compared with the lowest [71]. Again, the effect was only observed among smokers [71].

### Liver cancer

The World Cancer Research Fund/American Institute for Cancer Research (WCRF/AICR) report classifies the evidence for liver cancer as “limited-suggestive” [80]. In the joint analysis of liver cancer incidence in 12 cohorts according to recreational PA at baseline, the highest decile had a hazard ratio (HR) of 0.73 before taking BMI into account and decreased to a nonsignificant HR of 0.81 when BMI was included as a confounder [71].

### Esophageal cancer

There is limited but suggestive evidence of a protective effect of PA against esophageal adenocarcinoma and squamous cell carcinomas [81]. A meta-analysis found a risk ratio (RR) of 0.79 for esophageal adenocarcinoma and a nonsignificant RR of 0.94 for esophageal squamous cell carcinoma [82]. The pooled analyses of 1,44 million individuals from 12 cohorts showed an approximately 40% risk reduction in esophageal adenocarcinomas and a 24% risk reduction in esophageal squamous tumors for participants in the 90th percentile of leisure-time PA at baseline compared with the lowest PA group [71].

### Stomach cancer

The recent WCRF/AICR update still considers limited-not conclusive evidence available for stomach cancer [83]. A previous meta-analysis on gastric cancer estimated an RR of 0.82 for high versus low PA [82]. The pooled analysis of leisure-time PA at baseline in the 12 cohorts showed a 22% risk reduction for gastric cardia tumors when BMI was not taken into account, but stratification by BMI showed that the protective effect was only observed among overweight/obese people [71].

### Prostate cancer

There is a limited-not conclusive evidence of a link between PA and prostate cancer [84]. A systematic review showed substantial heterogeneity among 85 studies: 22 reported a statistically significant risk reduction, 25 reported a nonsignificant risk reduction, 31 did not find any association, and eight found an adverse effect of PA [85]. A higher incidence of prostate cancer (4% increase) was observed among the 10% more physically active participants in the pooled analysis of 12 cohort studies compared with those with a lower decile of activity. The authors hypothesized that this result could be due to a higher probability of prostate cancer screening in physically active men [71].

### Ovarian cancer

The evidence for ovarian cancer was considered limited-not conclusive [86]. While most case–control studies found significant risk reductions among very active women, most cohort studies failed to show a clear effect [87]. The pooling analysis of 1.44 million participants in 12 prospective cohorts in the USA and Europe did not find a protective effect of high leisure-time PA for this tumor [71]. The Nurses’ Health Study, a prospective cohort with updated information on leisure-time PA, revealed an increased risk for both low and high levels of premenopausal PA, while no association was observed in postmenopausal women [88].

### Pancreatic cancer

The WCRF/AICR report considers limited-not conclusive evidence for pancreatic cancer [89]. A meta-analysis yielded a statistically significant RR of 0.89 for high versus low PA [90]. Stronger effects were observed in case–control studies and for younger populations [90]. The pooling analysis of 12 cohort studies showed a non-statistically significant reduction of 5% in the most active group at baseline, but this effect was no longer observed when BMI was considered [71]. The EPIC-Norfolk cohort communicated a decreased risk in the highest category of total PA among participants younger than 60 years independent of BMI, while no effect was observed in older people [91].

### Kidney cancer

The WCRF/AICR panel considers limited-not conclusive evidence for kidney cancer [92]; however, a meta-analysis in 2013 estimated a 12% risk reduction in the high PA group that was stronger when combining only high-quality studies [93]. The pooled analysis of the 12 cohorts showed a risk reduction of 16% independent of BMI among the most active group [71].

### Bladder cancer

The evidence for bladder cancer is judged as limited-not conclusive [94]. A meta-analysis showed an RR of 0.85 for high versus low PA [95]. Moreover, the joint analysis of 12 prospective cohorts found a significantly reduced risk of bladder tumors in participants for the highest decile of leisure-time PA at baseline (HR = 0.88) [71].

### Other tumors

A systematic review and meta-analysis on PA and hematologic cancers showed a reduced risk for non-Hodgkin lymphoma and nonsignificant results for multiple myeloma and leukemias [96]. The pooled analysis of 12 cohorts found a protective effect of PA against myeloid leukemia, myeloma and head–neck carcinomas [71]. Interestingly, malignant melanomas were more frequent in participants at the highest decile of leisure-time PA, a finding attributed to greater sun exposure due to outdoor activity and an increased risk of sunburn [71].

### Summary and future directions

PA clearly reduces the risk of colon, BC and endometrial cancer. Furthermore, recent epidemiological studies suggest a protective effect for most cancer sites.

There is no conclusive evidence regarding the amount of PA needed to significantly reduce cancer risk, although it is likely tumor dependent.

New devices that routinely collect information on PA may help to increase the accuracy of PA measures and reduce information bias.

## Effect of physical activity on the prognosis of cancer

Several reviews and meta-analyses of observational studies have suggested the benefit of PA on cancer outcomes. Most of the studies included breast cancer (Tables 1, 2) and colon cancer survivors (Table 3). A few studies have been conducted on patients with other types of neoplasms, such as prostate (Table 4), esophageal, lung and kidney cancer (Table 5). In these studies, PA is reported as lifetime PA in the latest years before or after diagnosis. The outcomes reported are usually overall survival, cancer-related survival, cancer recurrence and quality of life (QoL).

**Table 1:** Meta-analysis of observational and interventional studies on the impact of exercise on breast cancer outcome

References	Population	PA	Outcome	Results
Lahart et al. [97]	123,574 BC survivors 1994–2014 Most studies observational	Pre-diagnosis	All-cause mortality	HR 0.82 (95% CI 0.75–0.96)
			BC mortality	HR 0.73 (95% CI 0.54–0.98)
			BC events	HR 0.72 (95% CI 0.56–0.91)
		After diagnosis	All-cause mortality	HR 0.52 (95% CI 0.43–0.64)
			BC mortality	HR 0.59 (95% CI 0.45–0.78)
			BC events	HR 0.79 (95% CI 0.63–0.98)
Lahart et al. [98]	5761 BC survivors from 63 randomized trials PA intervention	After diagnosis	All-cause mortality	No data
			BC recurrence	No data
			HRQoL, emotional function, perceived physical function, anxiety, and cardiorespiratory fitness	Small to moderate improvement

BC, breast cancer; HRQoL, health-related quality of life; PA, physical activity

**Table 2:** Summary of prospective observational studies on physical activity and prognosis in breast cancer patients

Study/references	Population	LTPA	Outcome	Results
Holmes et al. [99] Nurses’ Health Study	2987 Nurses with stage I-III BC, 1984–1998	After diagnosis ≥ 9 MET-h/week	BC-specific mortality	HR 0.50 (95% CI 0.34–0.74)
Irwin et al. [100] HEAL Study	933 Women with BC 1995–1998	Pre-diagnosis ≥ 9 MET-h/week	Overall survival	HR 0.69 (95% CI 0.45–1.06)
		After diagnosis ≥ 9 MET-h/week	Overall survival	HR 0.33 (95% CI 0.15–0.73)
Bao et al. [101] Shanghai BCSS	518 Women with TNBC	After diagnosis ≥ 7.6 MET-h/week or ≥ 2.5 MET-h/week	BC-specific mortality	HR 0.58 (95% CI 0.39–0.86)
			BC recurrence	HR 0.67 (95% CI 0.46–0.96)
Schmidt et al. [102] Germany	3393 Women with early BC 50–74 year	Pre-diagnosis ≥ 42 MET-h/week	All-cause mortality	HR 0.66 (95% CI 0.47–0.92)
			BC mortality	HR 0.80 (95% CI 0.53–1.21)
			Cancer recurrence	HR 0.65 (95% CI 0.44–0.97)
Holick et al. [103] Florida-Boston	4482 Invasive BC 1998–2001	After diagnosis ≥ 21 MET-h/week	BC mortality	HR 0.51 (95% CI 0.29–0.89)
			All-cause mortality	HR 0.44 (95% CI 0.32–0.60)
Ammitzboll et al. [104] Danish Diet, Cancer and Health Cohort	959 BC survivors	After diagnosis ≥ 8 MET-h/week	All-cause mortality	HR 0.67 (95% CI 0.47–0.99)
Friedenreich et al. [105] Canadian	1233 BC survivors 1995–1997	Pre-diagnosis 46.9 MET-h/w	BC mortality	HR 0.56 (95% CI 0.38–0.82)
			BC recurrence	HR 0.66 (95% CI 0.48–0.91)
Sternfeld et al. [106] LACE Study	Multivariable 1970 BC survivors	PA 6 months prior to diagnosis	BC mortality	No association confirmed
			BC recurrence	No association confirmed
			All-cause mortality	HR 0.66 (95% CI 0.42–1.03)
Irwin et al. [107] Women’s Health Initiative	4643 BC (in situ + invasive)	Prior to diagnosis ≥ 9 MET-h/week	All-cause mortality	HR 0.61 (95% CI 0.44–0.87)
		After diagnosis ≥ 9 MET-h/week	BC mortality	HR 0.61 (95% CI 0.43–0.99)
			All-cause mortality	HR 0.54 (95% CI 0.38–0.79)
Bertram et al. [108] WHEL Study	2361 Women with stage I-III BC	Baseline active	All-cause mortality	HR 0.47 (95% CI 0.26–0.84)
			BC events	No effect
		Adherence to activity guidelines after 1 year post-diagnosis	All-cause mortality	HR 0.65 (95% CI 0.47–0.91)
			BC events	No effect
Bradshaw et al. [109] Long Island BC Study	1033 BC (in situ + invasive) 1995–1996	After diagnosis ≥ 9 MET-h/week	All-cause mortality	HR 0.33 (95% CI 0.22–0.48)
			BC mortality	HR 0.27 (95% CI 0.15–0.46)

BC, breast cancer; LTPA, leisure-time physical activity; MET-h/week, metabolic equivalent task hours per week; TNBC: triple-negative breast cancer

BC events: BC progression, new primary BC, recurrence of BC

**Table 3:** Summary of observational studies on physical activity and prognosis in colorectal cancer patients

Study	Population	LTPA	Outcome	Results
Walter et al. [110]	3121 CRC patients	Latest LTPA ≥ 56 MET-h/week	Overall mortality CRC mortality	HR 0.75 (95% CI 0.61–0.91) HR 0.81 (95% CI 0.64–1.02)
Arem et al. [111] AARP Diet and Health Study	3797 CRC patients 1759 CRC patients	Pre-diagnosis LTPA > 7 MET-h/week Post-diagnosis LTPA > 7 MET-h/week	Overall mortality Overall mortality	HR 0.80 (95% CI 0.68–0.95) HR 0.69 (95% CI 0.49–0.98)
Meyerhardt et al. [112] CALGB 89803	832 Patients with stage III CRC	Post-diagnosis LTPA > 18 MET-h/week	Disease-free survival	HR 0.51 (95% CI 0.26–0.97)
van Blarigan et al. [113] CALGB 89803	992 Patients with stage III colon cancer	Post-diagnosis LTPA ≥ 8.75 MET-h/week	Overall survival	HR 0.64 (95% CI 0.45–0.92)
Meyerhardt et al. [112] Nurses’ Health Study	57 Women with stage I-III CRC	Post-diagnosis LTPA > 18 MET-h/week	CRC mortality Overall mortality	HR 0.39 (95% CI 0.18–0.82) HR 0.43 (95% CI 0.25–0.74)
Campbell et al. [114]	2293 Patients with stage I-III CRC	Pre-diagnosis LTPA ≥ 8.75 MET-h/week Post-diagnosis LTPA ≥ 8.75 MET-h/week	All-cause mortality All-cause mortality	RR 0.72 (95% CI 0.58–0.89) RR 0.58 (95% CI 0.47–0.71)

CRC, colorectal cancer; LTPA, leisure-time physical activity; MET-h/week, metabolic equivalent task hours per week

**Table 4:** Summary of observational studies on physical activity and prognosis in prostate cancer patients

Study	Population	LTPA	Outcome	Results
Richman et al. [115]	*N* = 1455 Non-metastatic PC	Walk briskly ≥ 3 h/week	Rate of progression	HR 0.43 (95% CI 0.21–0.91)
Friedenreich et al. [116]	*N* = 830 Stage II–IV PC 1997–2000	Post-diagnosis total activity > 119 MET-hours/week	All-cause mortality	HR 0.58 (95% CI 0.42–0.79)
		Pre- and post-diagnosis activity	PC mortality	HR 0.56 (95% CI 0.35–0.90)
		> 18 MET-hours/week	All-cause mortality	HR 0.66 (95% CI 0.49–0.88)
Kenfield et al. [117] Health Professional Follow-up Study	*N* = 2705 Non-metastatic PC 1990–2008	Post-diagnosis walking ≥ 90 min per week	All-cause mortality	HR 0.54 (95% CI 0.41–0.71)
		Post-diagnosis walking ≥ 3 h per week or vigorous activity	All-cause mortality	HR 0.51 (95% CI 0.36–0.72)

LTPA, leisure-time physical activity; MET-h/week, metabolic equivalent task hours per week; PC, prostate cancer

**Table 5:** Prospective observational studies on physical activity and prognosis in other cancers

Study	Population	LTPA	Outcome	Results
Liss et al. [118] Texas and San Diego	222,163 Kidney cancer survivors 1998–2004	Any PA	Kidney cancer-specific mortality	HR 0.50 (95% CI 0.27–0.93)
Sloan et al. [119] Rochester, US	1466 Lung cancer survivors 1997–2009	Physically active	Recurrence rate Overall survival	81% versus 82% (*P* = 0.62) 8.4 year versus 4.4 year (*P* < 0.0001)
Wang et al. [120] Chinese	303 Early esophageal cancer survivors	After surgery > 9 MET-h/week	All-cause mortality Risk of recurrence	HR 0.67 (95% CI 0.48–0.92) HR 0.31 (95% CI 0.22–0.43)

HR, hazard ratio; LTPA, leisure-time physical activity; MET, metabolic equivalent; PA, physical activity

Epidemiologic and observational studies show a decrease in the risk of cancer recurrence and all-cause mortality in patients who practice regular PA [121–123]. A systematic review of studies published through June 2013 concluded that PA performed before or after cancer diagnosis is associated with a reduced mortality risk among BC and colorectal cancer survivors [124]. Mortality in adult survivors of childhood cancer was inferior in those patients who practiced vigorous exercise after diagnosis in a large multicentric observational study [125]. In 2015, Lahart et al. [97] published a meta-analysis of 22 studies analyzing the impact of PA on BC outcomes. A literature search was performed using PubMed, EMBASE and CENTRAL databases from 1995 to October 2014. In 40% of the observational studies, the risk of relapse and death in BC survivors decreased in most physically active women. Most studies included an analysis of leisure-time PA and only a few of interventional PA programs. The majority of the studies did not perform a multivariable analysis to exclude the effect of known confounding factors, and less than half included clinical prognostic factors, such as stage, nodal status, age or type of treatment. These studies found a positive impact on all-cause and BC mortality in patients who practiced moderate or intense lifetime PA before the diagnosis of BC and in recent years before diagnosis. However, the authors recommend interpreting these results with caution due to the large heterogeneity of the studies. A post-diagnosis activity of at least 10 MET-hours/week was associated with a decrease in all-cause, and BC mortality and was not influenced by the heterogeneity; however, not all the studies could corroborate a decrease in recurrence risk. The “After Breast Cancer Pooling Project” included more than 13,000 women from four prospective cohorts of BC survivors in the USA and Shanghai and analyzed the association between PA at 18–48 months after diagnosis and risk of all-cause and BC-specific mortality and BC recurrence [126]. BC mortality was reduced in patients who achieved 18.7 or more MET-hours/week, and no association was found between PA and BC recurrence [126]. A comprehensive review of sixty-three interventional studies on women after BC adjuvant therapy concluded that PA interventions might have certain beneficial effects on QoL, cardiorespiratory fitness and psychological and social functions, but conclusions about BC recurrence, BC mortality and all-cause mortality could not be made [98].

Several prospective observational studies and meta-analyses in patients with colorectal cancer have suggested the benefit of PA before and after diagnosis in terms of improvements on all-cause and cancer-specific mortality after controlling for other confounding factors, such as BMI, sex, number of positive lymph nodes, age, baseline performance status (PS), adjuvant chemotherapy regimen or recurrence-free survival period [110, 111, 127, 128]. Similarly, three observational prospective studies in prostate cancer found a strong inverse relationship between exercise and the risk of cancer progression regardless of other known prognostic factors [115–117]. A Chinese study in patients who underwent esophagectomy for esophageal cancer supported the benefit of PA (> 9 MET-hours/week) on recurrence risk and all-cause mortality [120]. Data from a prospective observational study in kidney cancer survivors investigating PA and diet changes suggested a decrease in the recurrence rate in patients who did any PA compared with those that were totally inactive [118]. The only study in lung cancer survivors showed better overall survival in patients who met > 9 MET-hours/week, but no difference in the recurrence rate was observed [119].

In conclusion, the real impact of PA on the risk of relapse and cancer mortality is not well-defined. PA may contribute to reduced cancer-related mortality and all-cause mortality in cancer survivors by modifying fat accumulation and improving cardiovascular and skeletal muscle function [129]. Numerous prospective observational studies consistently showed the benefit of PA on cancer outcomes; however, most of these studies were based on measures from self-reported questionnaires, including heterogeneous populations, and only a few performed a multivariable analysis to exclude the contribution of other confounding factors. Interventional studies with reliable and objective measures of PA in homogeneous populations are needed to confirm the data from observational studies and to evaluate the real effect of exercise on cancer prognosis.

## Exercise-oncology: a pragmatic point of view for clinicians

Exercise-oncology is a new field of cancer care with the goal of the appropriate and rationale introduction of exercise programs into the overall management of cancer patients to take advantage of the numerous benefits associated with PA. Several major comprehensive cancer centers have created exercise-oncology units to implement these programs in a timely and organized manner. A collaborative work among rehab specialists, physiotherapists and exercise physiologists, as well as oncologists and radio-oncology specialists, is developed in these units.

Exercise has demonstrated numerous benefits on the QoL of patients with cancer throughout the history of the disease, ameliorating the negative impact of cancer on physical and psychological health and having a positive impact on patient survival [130–133].

Despite these benefits, many questions about PA/exercise in cancer patients remain, as it is particularly challenging to elucidate how much exercise is needed to achieve patient improvements and how the exercise should be recommended and monitored by clinicians.

### General PA/exercise recommendations for cancer patients

In 2010, the first exercise guidelines were published by a roundtable of the American College of Sports Medicine (ACSM) based on general WHO PA guidelines to the general population. These guidelines consist of a minimum exercise recommendation: 150 min of moderate-intensity exercise in 3–5 days combining 2 days of resistance exercise and 3 days of aerobic exercise or 70 min of high-intensity exercise combining 1 day of resistance exercise and 2 days of aerobic exercise [133].

### Exercise in the cancer treatment continuum

First, it is important to highlight that exercise is feasible, effective and safe in patients with cancer throughout the course of the disease. However, there are specific recommendations for the different moments of the disease and its therapies.

#### Presurgical exercise

It has been shown that presurgical high-intensity interval training in cancer patients is feasible and effective in improving cardiorespiratory fitness, which is typically measured as peak oxygen uptake (VO_2peak_); this training makes sense when patients need to achieve a specific VO_2peak_ to undergo surgery, as noted for patients with lung cancer [134]. The intervention was based on high-intensity aerobic exercise (cycling) from 50 to 100% of VO_2peak_ for 30 min, 5 days per week.

In another study in patients with BC, a presurgical intervention consisting of 180 min of moderate aerobic exercise and 40 min of strength training per week was associated with physiological changes and alterations in gene expression in tumor tissue (notably, downregulation of pathways related to cell cycle, RNA transport and DNA replication) [135].

#### Exercise during chemotherapy

Several studies using PA concomitantly with neoadjuvant and adjuvant chemotherapy have been performed with different approaches, demonstrating safety, effectiveness and fitness improvements [136].

Exercise programs concomitant with neoadjuvant chemotherapy are usually focused on improving the VO_2peak_ level or maintaining it at the baseline range after cancer treatment. Interventions are based on at least 3 days per week with different durations (from 4 to 12 weeks) in 30- to 60-min session with variable intensities, which range from 55 to 60% of VO_2peak_ at the start to 70–100% of VO_2peak_ at the end [137].

Exercise interventions concomitant with adjuvant therapy must take into account a safe starting time to be sure that surgical wounds are completely scarred. Different reviews have shown that exercise improves fitness capacity [138] and might reduce some cancer-related side effects, such as fatigue [136]. However, interventions in these studies were heterogeneous and did not often describe the intensity or type of exercise used. A recent meta-analysis suggested that a workload of 600 MET (intensity-minutes) was associated with a clinically significant improvement in fitness capacity, suggesting that a 10-week program of 90 min/week of supervised training at 70% of VO_2peak_ may be sufficient [139–141]. Another meta-analysis found that cancer survivors who completed 15 MET-h/week presented a 27% lower risk of cancer mortality with respect to controls, and this effect was greater in patients who were sedentary at pre-diagnosis (35% lower risk) [142].

Despite the aggressiveness of cancer therapies, medium to high-intensity exercise and different types of exercise interventions are well-tolerated by most patients. Both previous reviews mentioned above focused on exercise intervention during neoadjuvant and adjuvant treatments, including high-intensity intervention [137, 143].

In addition, resistance training has been shown to be safe and effective in preventing lean body mass loss and reducing body fat mass during neoadjuvant and adjuvant treatments [144].

#### Exercise in cancer survivors

It is well-known that cancer survivors obtain an improvement in QoL, body composition and physical fitness with exercise [130, 131, 144–146]. Again, the challenge in this population is to determine how much exercise is needed to achieve the maximum benefits. Related to exercise intensity, Gil-Rey et al. [147] showed that cancer survivors have an important reduction in their fitness capacity after cancer therapy and therefore suggest a reduction in exercise intensity at the beginning of training (i.e., 41–64% of VO_2max_). However, high-intensity training is feasible, safe and effective for cancer patients, and a shorter time of training is likely sufficient to obtain benefits, which should be taken into account in the implementation of exercise strategies [148].

More research is needed to determine the dose–response relationship between exercise and physical improvements given that some data from past clinical studies have suggested that an exercise intensity higher than that included in the general WHO recommendations might be needed to improve patients’ health status [147].

#### Exercise in patients with advanced and metastatic disease

Previous studies and reviews have shown that exercise is a safe and effective tool to improve fitness and functional capacity, strength, QoL and fatigue. Fitness and functional capacity were assessed by the VO_2peak_ and 6-min walking tests, showing significantly better results compared with the control group. In these studies, the aerobic exercise intensity ranged from 55 to 75% of VO_2peak_ [15, 16, 19, 29]. Muscle strength was assessed with the one-repetition maximum (1RM) or estimated 1RM test (lower and upper limbs), and exercise intensity in these studies ranged from 40 to 80% of 1RM. Program durations ranged from 5 to 12 weeks [22, 25]. With respect to body composition, significant changes were observed in lean mass, but no changes in fat mass, body mass or BMI were observed in previous studies. The low intensity of the exercise intervention (from 55 to 70% of VO_2peak_) might be a reason for these inconsistent results [15, 16, 33].

### Exercise supervision

**Fig. 1:**
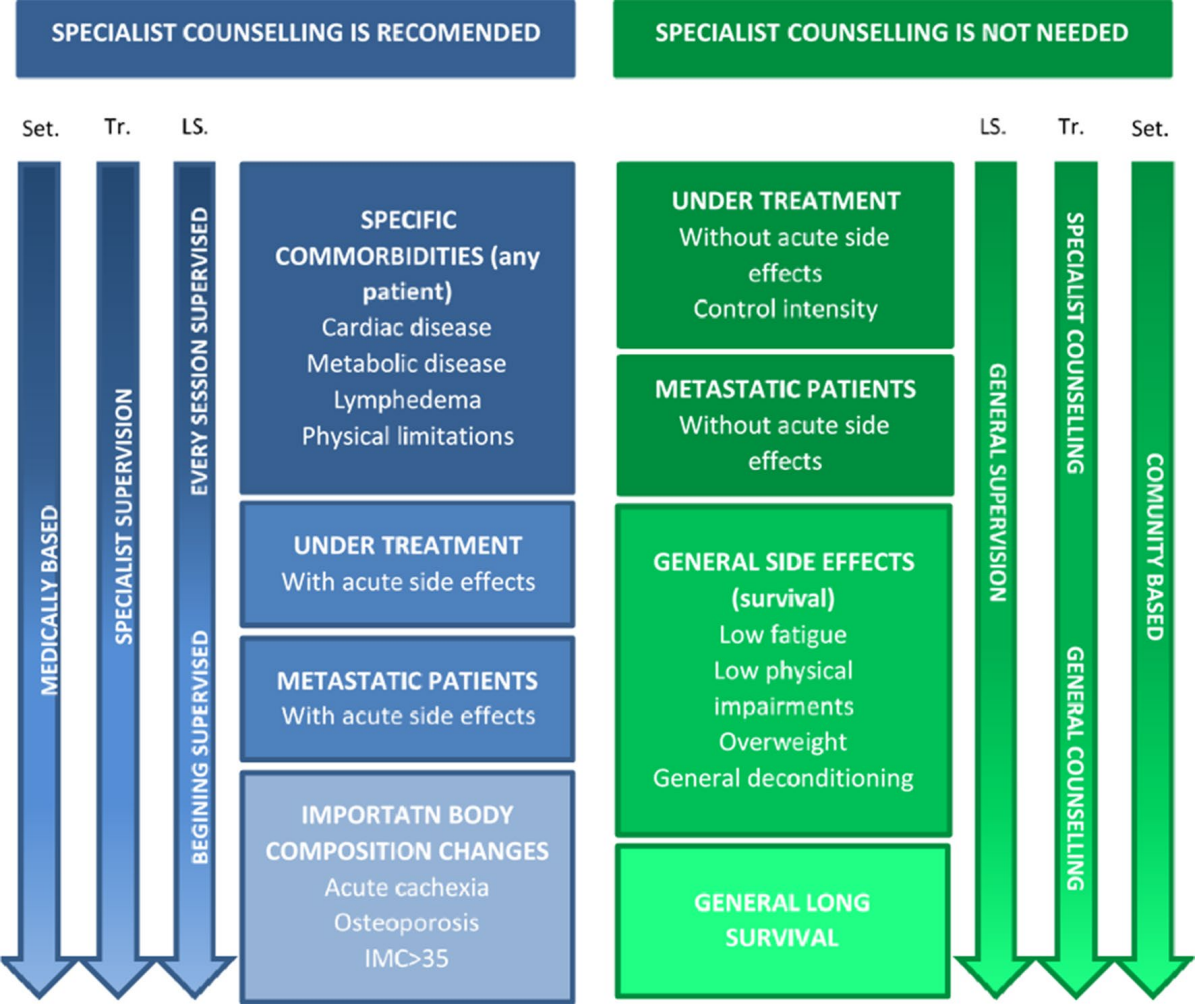
Distinguishing between patients who need specialist counseling and those who do not. Adaptation to the triage model for population-based screening of cancer survivors for weight management and physical activity interventions. Modified from National Academies of Sciences, Engineering, and Medicine 2018 [149]. Set. = setting; Tr. = training of professional; LS. = level of supervision. Specialist refers to clinicians, physical therapists, occupational therapists, dieticians, and clinical exercise physiologists

Although the benefits of exercise are well-established, the exercise dose–response and the best type of exercise in terms of duration and intensity remain unclear, making it difficult to establish how to provide specific recommendations to each individual patient and how to supervise the patient’s exercise by clinicians. With these caveats in mind, it might be wise to differentiate between patients who clearly need specialist counseling (as those under active treatment or metastatic patients, and all patients with side effects who limit them physically) and patients who do not (survival with limited side effects) (Fig. 1). An exercise-oncology specialist is an exercise professional with a previous background that includes a general qualification in exercise and health with specific knowledge in oncology items. Related to the oncology items, general knowledge about cancer biology, biomarkers and treatments and their side effects should be used to adapt to and individualize exercise to patients’ needs.

#### Challenges for patients: general supervision

For those patients who do not present the need for specialist counseling, the control of patients by informed clinicians could be sufficient to achieve reasonable results. In this respect, there are some specific guidelines that could be followed by patients and supervised by a nonspecialist with the help of different tests, devices or scales. For example, following WHO/ACSM guidelines or achieving more than 10,000 steps per day [150] are reasonable goals for cancer survivors (Table 6).

**Table 6:** General challenges for patients without specialist counseling needs based on existing guidelines

Recommendation	Challenge	Intensity
WHO/ACSM guidelines	150 min per week 30 min/3 times “aerobic” exercise 30 min/2 times strength exercises	Moderate
	75 min per week 25 min/2 times aerobic exercise 25 min/1 time strength exercises	High intensity
Survival recommendations [99, 107, 147, 151]	9 MET corresponding to 180 min of walking	5 km/h
Review psychological benefits [152]	12 MET; 90–120 min	Moderate intensity
Minimum step recommendations [150]	< 5000 steps/day	“Sedentary lifestyle index”
	5000–7499 steps/day	It is typical of daily activity excluding sports/exercise and might be considered “low active”
	7500–9999	“Somewhat active”
	≥ 10,000 steps/day	“Active”
	Individuals who take > 12,500 steps/day	“Highly active”

MET, metabolic equivalent

New technologies are improving methods to supervise the quality and quantity of exercise [153]. While behavioral interventions using text messages (with or without educational material and internet support) have produced limited effects on exercise adherence, mobile applications have been shown to be an effective and useful tool for both patients and providers to establish a healthy lifestyle. To achieve significant changes, it has been observed that these apps should include self-monitoring merged with other motivational techniques (goal setting, feedback on performance, review of goals, prompts, planning or barrier identifications, among others), allowing better supervision and control for patients and trainers [153].

#### Challenges for clinicians: learning exercise techniques

**Fig. 2:**
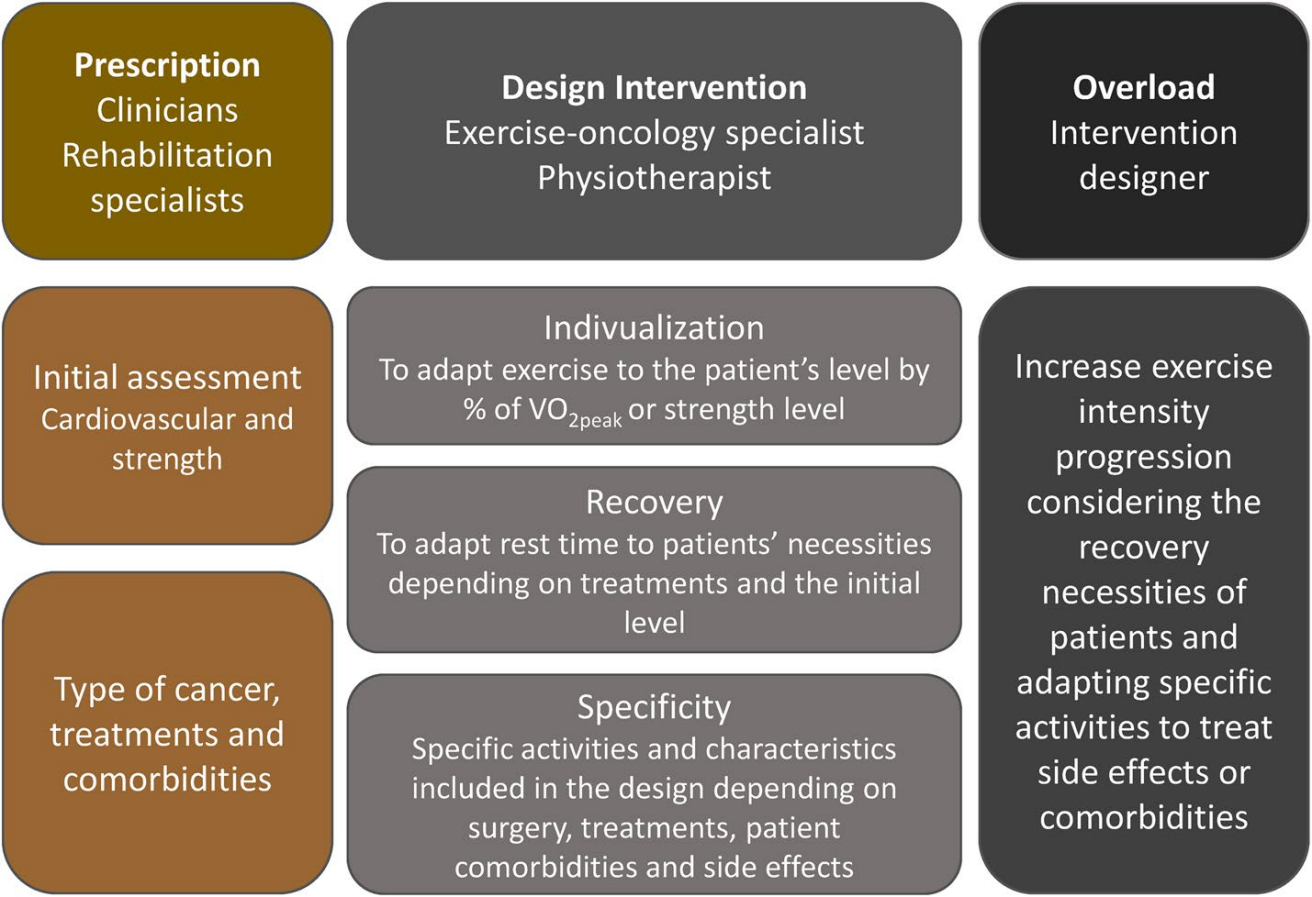
Specialists and activities that should be developed by each specialist to achieve an adequate exercise intervention for oncologic patients while taking into account training principles adapted to exercise-oncology

At present, exercise provides empowerment among health care providers, presenting a new challenge for them. One of the most important issues to address is who might prescribe and control exercise. It is possible that multidisciplinary committees, including oncologists, rehab departments and exercise physiologists, should be created to provide patients with the best counseling and training physicians to help individuals not requiring special help (Fig. 2).

However, while this scenario seems far away, other achievable proposals are feasible. The education of clinicians taking care of cancer patients and survivors about exercise techniques and control is a crucial point. Education and training should ideally start during university studies, although few institutions worldwide provide exercise theory and training to their future professionals. This education would have an impact not only in cancer patients but also on many other common pathologies such as cardiovascular, metabolic, joint and other diseases [154]. In accordance, patients’ associations playing a crucial role at present in providing exercise training and assistance until exercise will be included in the usual care.

### Future lines of research

Despite the increasing number of studies addressing the benefits of exercise for cancer patients and survivors in the last 5 years, further research remains essential to clarify many unanswered questions. The establishment of new, more concrete guidelines for exercise in cancer patients is necessary not only for the exercise-oncology specialist but also for oncologists and other clinicians who take care of cancer patients.

There is an urgent need to further clarify the biological mechanism that makes exercise an effective method of intervention to decrease cancer incidence and mortality and to improve overall health in cancer patients. Studies of the modification of biological biomarkers before and after exercise are crucial for understanding the underlying mechanisms through which exercise can exert its influence on cancer biology. Several preclinical studies (discussed above) and clinical studies of small sample sizes have provided preliminary evidence on the relevance of the immune system, cytokines and insulin-related pathways [155]; however, because the evidence is preliminary, larger and statistically powerful studies are required.

In addition, new studies aimed at identifying the optimal intensity and duration of PA are needed. The characteristics of cancer survivors differ from those of the healthy population to whom the recommendations of the different health organizations are directed.

The best method of introducing exercise into the lifestyle of patients is also a matter to be addressed. The most effective behavioral interventions to achieve long-term changes in a patient’s lifestyle must be defined, bearing in mind that cancer diagnosis and treatment are “learning moments” in which patients are willing to change their daily activities to improve their health. The feasibility of using new technologies, such as mobile health applications and wrist and watch bands, as well as interventions based on social networks should be investigated to favor adherence and motivation to these programs of adapted PA.

In addition, future research on intervention in metastatic cancer stages should be performed due to the lack of knowledge in this area and the potential interest in improving the tolerance and effectiveness of treatments and the QoL of patients, many of whom can live today for many years after relapse due to the effective and sustained disease palliation that can be achieved with modern systemic treatments.

## Conclusions

Regular PA is associated with major benefits to human health, including a reduced risk of some cancers.

The mechanisms through which exercise exerts its antitumor activity are still poorly understood but might be related to a direct effect on tumor cells (inhibition of tumor cell proliferation, induction of apoptosis, upregulation of tumor suppressor genes, anti-inflammatory effects) or to an enhancement of immune function.

There is convincing evidence that regular PA reduces the risk of colorectal cancer, while the reduction in postmenopausal BC and endometrial cancer risk is judged as probable. The effect of PA on the risk of other tumors is less evident but still possible.

Several epidemiological studies have suggested an association of regular PA with reduced cancer-related and all-cause mortality in some tumor types, particularly BC and colorectal cancer. The minimum amount of PA needed to achieve such a benefit is still unknown, although the US recommendations suggest that a minimum 10 MET-hours/week (equivalent to ≥ 150 min of moderate-intensity PA) is needed.

Exercise-oncology is a field of cancer care in which the goal is the introduction of exercise programs into the overall management of cancer patients. The first exercise guidelines for cancer patients were published in 2010 by the ACSM. These guidelines, which are mainly based on general WHO guidelines to the general population, consider that regular PA in cancer patients is safe and exerts positive effects in patients at multiple levels, particularly QoL. Exercise programs in cancer patients are feasible along the course of the disease, including the presurgical period, during adjuvant antitumor medical treatment (including chemotherapy) and in cancer survivors; a summary of these recommendations is shown in Table 7. However, the experience with regular exercise in metastatic cancer patients is limited.

**Table 7:** Exercise duration and intensity recommendation to cancer patients [147, 156]

Cancer treatment moment	Type of exercise	Description	Intensity	Duration (start with…)	Examples
Pre-surgery without other treatment	Endurance	High-intensity interval training	60–90% of VO_2peak_ [157, 158]	20–35 min	Walking Running Spinning
	Strength	Global strength exercises	Exercises with patient’s own body weight 0–40% of 1RM	2 sets of 10 repetitions	Global strength circuits
	Stretching	All body	Passive or active stretches	30 s/exercise	Yoga Stretching classes
Surgery/mammary reconstructions	Endurance	Light intensity, avoid pain	Depending on patient’ mobility [143]	20–40 min	Walking biking Avoid swimming
	Strength	Rehabilitation recommendations	Rehabilitation recommendations	Rehabilitation recommendations	Upper-limb surgery: light arm mobility and postural exercises Abdominal surgery: hypopressive abdominal exercises, and postural exercises—including isometric exercises
	Stretching	Rehabilitation recommendations	Rehabilitation recommendations	Rehabilitation recommendations	Passive stretching without pain, focusing on affected area, when specialist allows it
Under chemo/radiotherapy	Endurance	Adapted intervention to patient needs	From 41 to 64% (moderate) to 80–90% of VO_2peak_ (high intensity, if the patient was previously active)	3 days per week/20–35 min	Walking Dance Bike Spinning Running Avoid swimming
	Strength	Global strength to prevent sarcopenia or cachexia	From light movements to 40–60% of 1RM at first [159]. Depending on the patient’s comorbidities [143]	2 days per week, 2 sets of 10 repetitions	Yoga Pilates Elastic bands Global strength circuits Machines
	Stretching	Stretch gently all body. Special care with radiation areas. Avoid pain	Passive stretches	30 s/exercise	Yoga Stretching classes
Under hormone therapy	Endurance	Moderate to high intensity depending on the patient’s previous situation. Rest for 48 h after high-intensity training	From 41–64% (moderate) to 80–90% of VO_2peak_ (high intensity) if the patient was previously active	3 days per week/30–40 min	Walking Dance Bike Spinning Running Swimming
	Strength	Moderate to high intensity depending on the patient’s situation. Rest for 48 h after high-intensity training	From light movements to 40–60% of 1RM [159] at first. Depending on the patient’s comorbidities [143]	2 days per week, 2 sets of 10 repetitions	Yoga Pilates Elastic bands Global strength circuits Machines
	Stretch	All body	Passive or active stretches	30 s/exercise	Yoga Stretching classes
Survivors	Endurance	Moderate to high intensity depending on previous patients’ situation	From 41 to 64% (moderate) to 80–100% of VO_2peak_ (intense) if the patient was initially active	3 days per week/30–40 min	Walking Dance Bike Spinning Running Swimming
	Strength	Moderate to high intensity depending on the patient’s previous situation. Special care for patients with functional limitations. Avoid pain.	From light movements to 40–60% of 1RM [159] at first. Depending on the patient comorbidities [143]	2 days per week, 2 sets of 10 repetitions	Yoga Pilates Elastic bands Global strength circuits Machines
	Stretch	All body	Passive or active stretches	30 s/exercise	Yoga Stretching classes

RM, repetition maximum; VO_2peak_, peak oxygen uptake

The introduction of exercise programs into the global management of cancer patients remains a challenge due to conceptual and logistic issues. The most effective behavioral interventions to achieve long-term changes in a patient’s lifestyle must be defined. New technologies, such as mobile health applications and wrist and watch bands (the so-called “mHealth”), can be of great help to monitor the compliance to these programs. The optimal intensity and duration of PA should be defined with more precision in future studies. Regarding logistics, the intervention of both exercise-oncology specialists and trained clinicians is probably necessary at different time points to provide the best care. Several major comprehensive cancer centers have created exercise-oncology units to implement these programs in a timely and organized manner, and these models could serve as a reference for other institutions.
